# Effect of *CIMicifuga racemosa* on metaBOLIC parameters in women with menopausal symptoms: a retrospective observational study (CIMBOLIC)

**DOI:** 10.1007/s00404-019-05366-8

**Published:** 2019-11-16

**Authors:** Lena Friederichsen, Sabine Nebel, Catherine Zahner, Lukas Bütikofer, Petra Stute

**Affiliations:** 1 Department of Obstetrics and Gynecology, Spital Interlaken (hospitals fmi AG), Interlaken, Switzerland; 2 Max Zeller Söhne AG, Romanshorn, Switzerland; 3 CTU Bern, Institute of Social and Preventive Medicine (ISPM), University of Bern, Bern, Switzerland; 4 Department of Obstetrics and Gynecology, Inselspital, Bern University Hospital, University of Bern, Bern, Switzerland; 5 Gynaecologic Endocrinology and Reproductive Medicine, University Women’s Hospital, Theodor-Kocher-Haus, Friedbühlstrasse 19, 3010 Bern, Switzerland

**Keywords:** Black cohosh, *Cimicifuga racemosa*, Menopause, Body weight, HOMA index, Menopausal hormone therapy

## Abstract

**Purpose:**

To compare the influence of *Cimicifuga racemosa* extract (CR, Ze 450) and menopausal hormone therapy (MHT) on metabolic parameters and body weight in symptomatic menopausal women.

**Methods:**

In this monocentric retrospective cohort study, women over 40 years old with a first consultation between 2009 and 2016 were screened. Included in the final analysis were women treated with either MHT or CR and having at least one follow-up consultation. Metabolic serum parameters (lipids, glucose, insulin, and HOMA-IR), body weight, and menopausal symptoms [Menopause Rating Scale (MRS)-II] were the main outcome measures. Statistical analysis by uni- and multi-variable linear mixed-effects regression models assuming a linear effect of time.

**Results:**

174 women were included in the final analysis (CR *n* = 32, MHT *n* = 142). There was no difference between the groups regarding baseline characteristics (age, BMI, serum metabolic parameters, hormones, and blood pressure) and total MRS-II score, while reproductive stage differed significantly with more postmenopausal women treated with CR (83%) than MHT (55%) (*p* = 0.038). Median follow-up time was 12 months. In both groups, metabolic serum parameters and body weight did not change over the follow-up period, while total and MRS-II subscores improved.

**Conclusion:**

Menopausal symptoms improved significantly in both groups (MHT and CR), while serum metabolic parameters and body weight did not change in MHT- or CR-treated women.

**Electronic supplementary material:**

The online version of this article (10.1007/s00404-019-05366-8) contains supplementary material, which is available to authorized users.

## Introduction

The menopausal transition and ageing are accompanied by body weight increase of about 0.5 kg annually [1–3]. Weight gain, especially during menopause, has been significantly associated with increased risk of several diseases [2]. Furthermore, body composition changes with visceral fat depots increasing and lean body mass decreasing [4] often accompanied by adverse changes in metabolic parameters [5]. Menopausal hormone therapy (MHT) is usually indicated for menopausal symptom relief, but also has a positive impact on body weight, body composition, and metabolic parameters [6–8].

Black cohosh (*Cimicifuga racemosa*, CR) is an alternative treatment for menopausal hot flushes [9, 10]. Its precise mechanism of action is controversial. For hot flush reduction, CR is thought to modulate the neurotransmitter signaling pathways in the brain and not to exert estrogenic effects [11–13]. In vitro, the Black cohosh dry extract Ze 450 [(DER 4.5–8.5:1), extraction solvent ethanol 60% (V/V)] has been shown to dose-dependently activate AMP-activated protein kinase (AMPK), an activity regulator of different key enzymes involved in lipid synthesis (HMG-CoA reductase and acetyl-CoA carboxylase), and some glucose transporters (Glut 1/4) [14]. Herein, AMPK activation was more pronounced with Ze 450 than metformin treatment. These findings were confirmed in ob/ob mice showing a significant reduction of water intake and a normalization of the glucose and insulin response to a glucose challenge test [14]. Thus, Ze 450 may improve metabolic control via improving insulin receptor sensitivity. The aim of this monocentric retrospective study was to compare the impact of the Black cohosh dry extract Ze 450 versus MHT on body weight and metabolic parameters, such as serum lipids, glucose, insulin, and HOMA-IR in symptomatic menopausal women.

## Methods

### Study design

CIMBOLIC is an investigator-initiated monocentric retrospective observational study. Coded health-related data of all patients above age 40 presenting for first-time consultation at the Menopause Centre of the Department of Obstetrics and Gynecology, Inselspital Bern, between 01.04.2009 and 31.12.2016 were abstracted from medical records. Routine assessment comprised personal and family history including reproductive age [15], medication, lifestyle factors (smoking, alcohol), questionnaire Menopause Rating Scale (MRS)-II [16], anthropological parameters (body weight, height, body mass index, blood pressure, and waist circumference), laboratory blood tests, and risk evaluation of certain non-communicable diseases (cardiovascular disease, breast cancer, osteoporosis), respectively (supplementary file 1). The selection of the treatment option (CR or MHT) was based on the above-described routine assessment by the physician, a benefit–risk evaluation, and the patients wish at the time of consultation. For the final analysis, all patients treated with either Ze 450 (Cimifemin^®^ forte = 13 mg dry extract, Cimifemin^®^ uno = 6.5 mg dry extract or Climavita^®^ forte = 13 mg dry extract) or any MHT (but not both) during the follow-up period and having at least one follow-up visit including blood tests were eligible. The study was approved by the cantonal ethics committee (Kantonale Ethik-kommission Bern (KEK) Switzerland No 2016-01179).

### Data sources

Data from medical records were transferred to a REDCap (Research Electronic Data Capture) database. Generation, transmission, storage, and analysis of health-related personal data within this project were according to the current Swiss legal requirements for data protection and were performed according to the Ordinance HRO Art. 5. This observational study is reported according to the STROBE statement (see www.strobe-statement.org).

### Statistical methods

Patients were grouped according to whether they were treated with Ze 450 or MHT during the follow-up period. Continuous and categorical variables are presented by group using median with lower and upper quartiles (lq, uq), and number and percentage of patients, respectively. Baseline characteristics between groups were compared using Wilcoxon rank-sum tests and Fisher’s exact tests for continuous and categorical variables, respectively. Linear mixed-effects regression models were used for the analysis of a change over time within and between groups. The endpoint values at baseline and each follow-up were used as dependent variable and the treatment group (i.e., MHT or Ze 450), the time, and the interaction of time and group as fixed explanatory variables. A random intercept was included for each patient to account for intra-patient correlations. The models were fitted with restricted maximum likelihood, the degrees of freedom were calculated using the Satterthwaite approximation and 95% confidence intervals (95% CI) based on the t distribution. Marginal effects for each group were calculated using Stata’s margin command and expressed as mean change per year with 95% CI and a *p* value. The difference between groups (i.e., the interaction term of group and time) was expressed as mean difference per year (Ze 450 minus MHT) with 95% CI and a *p* value. For the main analysis, all patients in the full analysis set were included, regardless whether a baseline and a follow-up assessment were available for a specific endpoint. For a sensitivity analysis, only patients with a baseline and at least one follow-up measurement were included (referred to as complete case analysis). We used a multi-variable linear mixed-effects regression to adjust for confounding. All baseline variables with a *p* value < 0.2 in the baseline comparison and less than 30% missingness in both groups were included as covariates. An overall control of the type I error rate was not done because of the explorative nature of the study. The reported *p* values were not corrected for multiplicity and have to be interpreted accordingly. All analyses were done with Stata [StataCorp. Stata Statistical Software: Release 14. StataCorp, College Station, TX: StataCorp LP., 2015.] or R [R Core Team. R: A Language and Environment for Statistical Computing. R Foundation for Statistical Computing, Vienna, Austria, 2016. URL http://www.R-project.org. ISBN 3-900051-07-0.].

## Results

### Participants

**Fig. 1:**
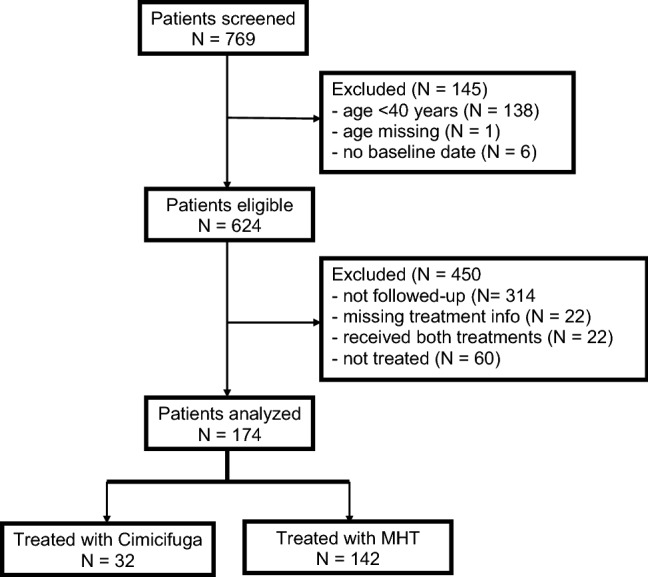
Patient flow from screening until final analysis

Figure 1 displays the patient flow. Of 769 patients screened, 174 were eligible for the final analysis. Of those, 32 were treated with Ze 450 and 142 with MHT, respectively. Current systemic MHT regimens comprised mainly estrogen–progestogen combinations (*n* = 89; 63%), while the remainder received either estrogens only, progestogens only, estrogen–androgen combinations, or tibolone, respectively. Only few patients in the Ze 450 group (*n* = 3; 9%) but most in the MHT group (*n* = 103; 73%) had a history of MHT use. However, at first consultation few patients were using Ze 450 (*n* = 8; 25%) or MHT (*n* = 49; 35%), respectively. All Black cohosh user had a Ze 450 product. Of those, the majority had a daily dosage of Ze 450 at 13 mg dry extract (*n* = 25; 78%; recommended dose 1 tablet daily).

### Subjects’ characteristics at baseline

Table 1 presents subjects’ anthropological, reproductive, and lifestyle parameters at baseline. There was no significant intergroup difference but for reproductive stage (*p* = 0.038). The majority of women treated with Ze 450 were postmenopausal (*n* = 24; 83%), while only one in two women treated with MHT fell into this category (*n* = 66; 55%). About one-third of MHT-treated women were either premenopausal (*n* = 21; 17%) or in the early menopausal transition (*n* = 23; 19%). In both groups, mean follow-up was 12 months (Ze 450 lq, uq 12.0, 24.0; MHT lq, uq 12.0, 36.0; *p* = 0.91). There was a significant difference in the number of follow-up visits (*p* = 0.011). 88% (Ze 450) and 61% (MHT) of subjects had only one follow-up visit. A few women treated with Ze 450 had two (*n* = 2; 6%), three (*n* = 1; 3%), or four (*n* = 1; 3%) follow-up visits, compared to 41 (29%) with two, 12 (8%) with three, and 3 (2%) with four follow-up visits in the MHT group.

**Table 1:** Subjects’ characteristics at baseline

Parameter	Ze 450 (*N* = 32)		MHT (*N* = 142)		*p* value
	*n*	Median [lq, uq] or *n* (%)	*n*	Median [lq, uq] or *n* (%)	
Age (years)	32	53.0 [48.5, 55.0]	142	52.0 [47.0, 55.0]	0.93
Body mass index (kg/m^2^)	31	24.5 [22.7, 27.4]	136	24.1 [21.2, 28.6]	0.68
Waist circumference (cm)	26	85.0 [79.0, 102.0]	122	88.0 [79.0, 96.0]	0.79
Systolic blood pressure (mmHg)	31	120 [112, 135]	138	120 [112, 130]	0.50
Diastolic blood pressure (mmHg)	31	80.0 [75.0, 88.0]	138	80.0 [70.0, 84.0]	0.13
Age at menarche (years)	31	14.0 [13.0, 15.0]	134	13.0 [12.0, 15.0]	0.16
Cycle length (days)	9	28.0 [28.0, 29.0]	24	28.0 [26.5, 28.0]	0.36
Age at menopause (years)	18	49.5 [45.0, 53.0]	67	48.0 [42.0, 51.0]	0.14
Time since last menstruation (years)	29	2.40 [0.47, 10.54]	124	2.42 [0.28, 8.44]	0.75
Reproductive stage according to STRAW + 10 [15]	29		121		0.038
Premenopause		1 (3%)		21 (17%)	
Early menopausal transition		2 (7%)		23 (19%)	
Late menopausal transition		2 (7%)		11 (9%)	
Postmenopause		24 (83%)		66 (55%)	
Smoking	32		142		0.09
Never		18 (56%)		102 (72%)	
Currently		8 (25%)		30 (21%)	
In the past		6 (19%)		10 (7%)	
Alcohol	32		142		0.67
Never		16 (50%)		59 (42%)	
Regularly		6 (19%)		27 (19%)	
From time to time		10 (31%)		56 (39%)	
AGLA® 10-year risk for fatal and non-fatal coronary heart disease (%)	21	0.70 [0.50, 1.20]	89	0.40 [0.20, 1.20]	0.12
FRAX® 10-year risk for osteoporotic fractures (%)	29	5.70 [4.40, 7.00]	127	5.60 [4.10, 7.10]	0.68
GAIL® 5-year risk for breast cancer (%)	31	1.30 [1.20, 1.50]	137	1.40 [1.10, 1.90]	0.22

*N* total number of observations, *n* number of non-missing observations

Table 2 presents the type and intensity of menopausal symptoms at baseline, assessed by the questionnaire MRS-II. In both groups, median intensity of overall menopausal symptoms (MRS-II total score) at baseline was moderate (defined as score 9–16) to severe (defined as score ≥ 17) (*p* = 0.46). When differentiating between menopausal symptom subdomains, both groups suffered from moderate vegetative symptoms (defined as score 5–8) (*p* = 0.10) and moderate (defined as score 2–3)-to-severe (defined as score ≥ 4) urogenital symptoms (*p* = 0.89), respectively. Psychological menopausal symptoms were moderate (defined as score 4–6) at baseline in both groups with women choosing MHT later on being slightly but significantly more affected than women choosing Ze 450 (*p* = 0.011).

**Table 2:** Prevalence of menopausal symptoms at baseline, assessed by MRS-II

MRS-II score	Ze 450 (*N *= 32)		MHT (*N *= 142)		*p* value
	*n*	Median [lq, uq]	*n*	Median [lq, uq]	
Total score (Q1–Q11)	25	16.0 [9.0, 20.0]	129	17.0 [11.0, 21.0]	0.46
Vegetative subscore (Q1, Q2, Q3, Q11)	26	8.00 [5.00, 10.00]	129	6.00 [4.00, 9.00]	0.10
Psychological subscore (Q4–Q7)	26	4.00 [2.00, 5.00]	129	5.00 [3.00, 9.00]	0.011
Urogenital subscore (Q8–Q10)	26	3.50 [2.00, 6.00]	129	4.00 [2.00, 6.00]	0.89

*N* total number of observations, *n* number of non-missing observations, *Q* question

### Change of menopausal symptoms, body weight, and metabolic parameters over time

We did not find any evidence for a within-group change in body weight, carbohydrate metabolism (glucose, insulin, and HOMA-IR), and serum lipids (total cholesterol, LDL-cholesterol, HDL-cholesterol, and triglycerides), neither in women treated with Ze 450 nor with MHT (Table 3). For MHT, we found a significant improvement over time of the MRS-II total score (− 0.99 [95% CI − 1.42, − 0.55] per year; *p* < 0.0001), MRS-II vegetative subscore (− 0.24 [95% CI − 0.45, − 0.03] per year; *p* = 0.023), MRS-II psychological subscore (− 0.48 [95% CI − 0.71, − 0.25]; *p* < 0.0001), and MRS-II urogenital subscore (− 0.28 [95% CI − 0.45, − 0.11]; *p* = 0.001). Similarly, treatment with Ze 450 significantly improved the MRS-II vegetative subscore (− 0.81 [95% CI − 1.57, − 0.04]; *p* = 0.039) and urogenital subscore (− 0.64 [95% CI − 1.26, − 0.01]; *p* = 0.045) (Table 3). MRS-II total score had the same trend (− 1.43 [95% CI − 3.16, 0.30]; *p* = 0.11). Between-group comparisons did not reveal significant differences for any endpoint (Table 3).

**Table 3:** Change of menopausal symptoms, body weight, and metabolic parameters over time

	*N* observations/patients	Ze 450		MHT		Difference (Ze 450-MHT)	
		Change per year (95% CI)	*p* value	Change per year (95% CI)	*p* value	Per year (95% CI)	*p* value
Body weight (kg)	239/171	− 0.17 (− 1.15, 0.82)	0.74	0.46 (− 0.02, 0.94)	0.06	− 0.63 (− 1.72, 0.47)	0.26
Fasting glucose (mmol/l)	208/145	0.06 (− 0.11, 0.23)	0.47	0.00 (− 0.08, 0.08)	0.94	0.06 (− 0.13, 0.25)	0.53
Fasting insulin (mU/l)	140/114	0.51 (− 0.99, 2.00)	0.51	− 0.26 (− 0.95, 0.44)	0.47	0.76 (− 0.89, 2.41)	0.37
HOMA index	133/110	0.17 (− 0.23, 0.57)	0.41	− 0.09 (− 0.27, 0.10)	0.36	0.26 (− 0.19, 0.70)	0.26
Total cholesterol (mmol/l)	232/157	0.07 (− 0.06, 0.19)	0.29	0.02 (− 0.05, 0.08)	0.65	0.05 (− 0.09, 0.19)	0.47
HDL-cholesterol (mmol/l)	232/157	0.01 (− 0.06, 0.07)	0.84	0.01 (− 0.03, 0.04)	0.74	0.00 (− 0.08, 0.08)	0.98
LDL-cholesterol (mmol/l)	231/158	0.04 (− 0.08, 0.15)	0.55	− 0.01 (− 0.08, 0.05)	0.65	0.05 (− 0.08, 0.18)	0.46
Triglyceride (mmol/l)	233/157	− 0.04 (− 0.17, 0.09)	0.58	0.00 (− 0.07, 0.07)	0.94	− 0.04 (− 0.19, 0.11)	0.59
MRS-II Total score	282/158	− 1.43 (− 3.16, 0.30)	0.11	− 0.99 (− 1.42, − 0.55)	< 0.0001	− 0.44 (− 2.23, 1.34)	0.63
MRS-II vegetative score	283/158	− 0.81 (− 1.57, − 0.04)	0.039	− 0.24 (− 0.45, − 0.03)	0.023	− 0.57 (− 1.36, 0.23)	0.16
MRS-II psychological score	283/158	0.28 (− 0.56, 1.13)	0.51	− 0.48 (− 0.71, − 0.25)	< 0.0001	0.76 (− 0.11, 1.63)	0.09
MRS-II urogenital score	283/158	− 0.64 (− 1.26, − 0.01)	0.045	− 0.28 (− 0.45, − 0.11)	0.001	− 0.36 (− 1.00, 0.29)	0.28

To adjust for potential confounding, we included baseline variables with a *p* value < 0.2 in the baseline comparisons and less than 30% missingness (diastolic blood pressure, age at menarche, reproductive stage, smoking status, MRS-II vegetative subscore, and MRS-II psychological subscore) in multi-variable models (Table 4). Results were similar as in the univariable models. For MHT, we found evidence for a change of the MRS-II total score (− 0.87 [95% CI − 1.26, − 0.48] per year; *p* < 0.0001), the psychological subscore (− 0.42 [95% CI − 0.60, − 0.24] per year; *p* < 0.0001), and the urogenital subscore (− 0.24 [95% CI − 0.42 to − 0.07] per year; *p* = 0.005). For Ze 450, we found some evidence for a change of the total score (− 1.82 [95% CI − 3.65, 0.02]; *p* = 0.05) and the urogenital subscore (− 0.79 [95% CI − 1.60, 0.02]; *p* = 0.05). We did not find any evidence for a between-group difference. Furthermore, we did not find within or between-group changes for body weight and serum metabolic parameter (total cholesterol, LDL-cholesterol, HDL-cholesterol, triglycerides, fasting glucose, insulin, and HOMA-IR) (Table 4).

**Table 4:** Change of body weight, metabolic parameters and menopausal symptoms over time after adjustment for baseline covariates (diastolic blood pressure, age at menarche, reproductive stage, smoking status, MRS-II vegetative subscore, and MRS-II psychological subscore)

	*N* observations/patients	Ze 450		MHT		Difference (Ze 450−MHT)	
		Change per year (95% CI)	*p* value	Change per year (95% CI)	*p* value	Per year (95% CI)	*p* value
Body weight (kg)	180/126	− 0.21 (− 1.23, 0.81)	0.69	0.35 (− 0.10, 0.79)	0.13	− 0.55 (− 1.67, 0.56)	0.33
Fasting glucose (mmol/l)	153/106	0.04 (− 0.18, 0.26)	0.72	− 0.00 (− 0.09, 0.09)	0.97	0.04 (− 0.19, 0.28)	0.73
Fasting insulin (mU/l)	105/85	1.12 (− 0.95, 3.20)	0.29	− 0.53 (− 1.37, 0.31)	0.21	1.66 (− 0.58, 3.89)	0.15
HOMA index	99/82	0.33 (− 0.24, 0.89)	0.26	− 0.13 (− 0.36, 0.10)	0.26	0.46 (− 0.15, 1.07)	0.14
Total cholesterol (mmol/l)	169/112	0.09 (− 0.04, 0.22)	0.17	0.00 (− 0.06, 0.07)	0.89	0.09 (− 0.06, 0.24)	0.25
HDL-cholesterol (mmol/l)	169/112	− 0.01 (− 0.08, 0.07)	0.84	− 0.00 (− 0.04, 0.04)	0.89	− 0.00 (− 0.09, 0.08)	0.91
LDL-cholesterol (mmol/l)	168/113	0.06 (− 0.07, 0.20)	0.34	− 0.01 (− 0.08, 0.05)	0.70	0.08 (− 0.07, 0.22)	0.30
Triglyceride (mmol/l)	169/112	0.01 (− 0.11, 0.13)	0.87	0.00 (− 0.06, 0.07)	0.94	0.01 (− 0.13, 0.15)	0.91
MRS-II Total score	231/126	− 1.82 (− 3.65, 0.02)	0.05	− 0.87 (− 1.26, − 0.48)	< 0.0001	− 0.94 (− 2.82, 0.93)	0.32
MRS-II vegetative score	231/126	− 0.69 (− 1.50, 0.12)	0.10	− 0.16 (− 0.33, 0.01)	0.07	− 0.53 (− 1.36, 0.29)	0.21
MRS-II psychological score	231/126	− 0.14 (− 1.00, 0.72)	0.75	− 0.42 (− 0.60, − 0.24)	< 0.0001	0.28 (− 0.59, 1.16)	0.52
MRS-II urogenital score	231/126	− 0.79 (− 1.60, 0.02)	0.05	− 0.24 (− 0.42, − 0.07)	0.005	− 0.55 (− 1.37, 0.28)	0.19

Only the effects of MHT and Ze 450 are shown

## Discussion

This monocentric, retrospective, observational study found that (1) both the Black cohosh dry extract Ze 450 and MHT markedly reduced menopausal symptoms over time without displaying significant intergroup differences with (2) MHT having a significant beneficial impact on psychological and urogenital symptom relief. Furthermore (3) in contrast to our expectations of weight gain during menopause [1, 2], we did not find any evidence for a change in body weight and metabolic parameters (serum lipids, carbohydrate metabolism) over time. However, uncertainty of the estimated effects was large. Finally, (4) potential confounders did not alter the results.

Clearly, this study has some limitations. The study was explorative and we tested many hypotheses without controlling the overall type I error rate—all results have to be interpreted accordingly. As this was a retrospective observational study, treatment indication, dosage, and duration were heterogenous. Sample size per group was small, the number of missing data was quite high, and we did not have an untreated control group. The power to detect differences between groups was small and not finding any does not provide evidence for the equality of the treatments. The pre-specified power analysis showed that 150 and 850 patients treated with Ze 450 and MHT, respectively, would be necessary to detect realistic effect sizes of 0.25 with a power of 80%. The absence of an untreated control group makes it impossible to exclude a placebo effect, in particular since we only found effects on subjective endpoints (the MRS scores). We assumed linear effects of time on all outcomes. This assumption is unlikely to be true but seems reasonable over the rather short follow-up time. The small sample size did not allow us to study the effect of time in more detail. On the other hand, the strength of the study was its real-life character when treatment initiation depends on multiple factors and does not exclude anyone like in prospective randomized-controlled trials. These results can, therefore, be transferred into the daily life situation of gynecologists working in outpatient clinics and private practices.

Interestingly, serum vitamin D3 levels were significantly lower in Ze 450 than in MHT-treated women. According to national guidelines, mean serum vitamin D3 levels were below the recommended serum levels within the Black cohosh cohort but within the recommended range in MHT-treated women (> 50 mmol/l) [17]. However, vitamin D supplementation has not been shown to have an impact on, e.g., glycemia or insulin resistance [18]. The assessment of vitamin D was incomplete (63% and 75% of the women had an assessment at baseline in Ze 450 and MHT group, respectively) and we did not include it in the multi-variable model.

The observation of a beneficial impact of the Black cohosh dry extract Ze 450 on menopausal symptoms is shown in an RCT [19] and supports a previous systematic review showing a reduction of vasomotor symptoms if Black cohosh products approved by regulatory authorities were applied (in contrast to Black cohosh products regulated as food supplementation) [10]. At baseline, menopausal symptoms were moderate to severe in both cohorts and improved over time with the Black cohosh extract Ze 450 and MHT being comparably effective. Interestingly, women with psychological menopausal symptoms were more likely to choose MHT later on. This might be due to the significantly increased risk of depression during perimenopause [20] and the known positive impact of MHT on mood [8, 21]. However, due to the small sample size, we were not able to differentiate between different MHT formulations, dosages, and indications. As significantly more women treated with Ze 450 than MHT were postmenopausal, we can only speculate that MHT was also prescribed for other indications than vasomotor symptom relief, e.g., for menstrual cycle regulation.

Both the menopausal transition and ageing have been shown to be accompanied by body weight increase and adverse changes in body composition and metabolic parameters [1, 2, 4, 5]. A study showed that women with metabolic syndrome had a higher total score of MRS and a higher subscale score for somatic symptoms, such as hot flashes and sweating [22]. For most affected women, any treatment with little adverse effects that may slow down or even stop this progression would be welcomed. For MHT, beneficial effects on body weight, body composition, and metabolic parameters have been described before [6–8]. However, many menopausal women and physicians are reluctant to use MHT due to the perceived risks, especially breast cancer risk [23]. Therefore, herbal medicinal products such as Black cohosh or phytoestrogen supplements have gained much attention in recent years. Just recently, a systematic review and meta-analysis reported a decrease in body weight with phytoestrogen supplementation in healthy postmenopausal women, but body weight gain in postmenopausal women with preexisting metabolic disorders [24]. In the present study, we did not observe a negative impact of Ze 450 on body weight gain and metabolic parameters in any subgroup. Yet, in general, women in our cohort were quite healthy. Thus, if a herbal product like Black cohosh dry extract Ze 450 had a stabilizing impact on body weight and metabolic parameters in menopausal women, the net benefit would outperform the rare potential, mostly short-term gastrointestinal, side effects. Our study in women supports previous findings showing a beneficial effect of Ze 450 on AMPK activity in vitro and on metabolic parameters in rats [14]. However, we can only speculate on the exact mechanism of action of Ze 450 in our study as we did not measure AMPK activity.

This is the first study in humans on the impact of Ze 450 on body weight and metabolic parameters. Definitely, these first results need confirmation in bigger and prospective trials.

## Conclusion

Both MHT and Black cohosh improved menopausal symptoms. Body weight and serum metabolic parameters did not change in MHT or Black cohosh-treated women.

## Electronic supplementary material

Below is the link to the electronic supplementary material.

Supplementary file 1 Overview of the assessed blood chemistry, personal and family history. (DOCX 18 kb)

## Data Availability

Data were not available as analysis for further publications is in preparation.
