# Supplementary material for: Effect of CIMicifuga racemosa on metaBOLIC parameters in women with menopausal symptoms: a retrospective observational study (CIMBOLIC)
Source: Arch Gynecol Obstet. 2019 Nov 16;301(2):517–23. doi: 10.1007/s00404-019-05366-8 (PMC7033259; doi:10.1007/s00404-019-05366-8)
Supplement: Supplementary file 1 — Overview of the assessed blood chemistry, personal and family history. (DOCX 18 kb) [file 404_2019_5366_MOESM1_ESM.docx]

Supplementary file 1: Personal and family medical history.

I Anthropological parameters

Age [years], body weight [kg], height [m], body mass index [kg/m^2^], systolic and diastolic blood pressure [mmHg], waist circumference [cm]

II Reproductive parameters

Age at menarche [years], cycle length [days], pregnancy [n], delivery [n], age at first delivery [years], time since last period [months], reproductive stage according to STRAW-10 [premenopause, early menopausal transition, late menopausal transition, postmenopause], age at menopause [years]

III Duration of former use of hormones

Contraception: oral combined hormonal contraception [years], oral progestin [years], non-oral progestin [years], hormonal intrauterine device [years];

Menopausal hormone therapy (MHT): estrogens only (ET) [years], estrogen-progestogen combination (EPT) [years], oral progestogen [years]

Others: selective estrogen receptor modulator (SERM) [years]

IV Personal and family medical history

Myocardial infarction, stroke, venous thromboembolism, cancer, diabetes mellitus

V Surgery

Hysterectomy, unilateral ovariectomy, bilateral ovariectomy, breast biopsy

VI Lifestyle

Smoking, alcohol

VII Menopause Rating Scale (MRS)-II

Total score, vegetative subscore, psychological subscore, urogenital subscore

VIII Risk calculators for chronic non-communicable diseases

AGLA® (Swiss Atherosclerosis Association): 10-years risk for fatal and non-fatal coronary heart disease

FRAX® (Fracture Risk Assessment Tool): 10-years risk for osteoporotic fracture

GAIL® (Breast Cancer Risk Assessment Tool of the National Cancer Institute): 5-years and lifetime risk for breast cancer

IX Laboratory blood test

Follicle stimulating hormone (FSH) [U/l], luteinizing hormone (LH) [U/l], LH/FSH ratio (calculated), prolactin (PRL) [mcg/l], estradiol (E2) [pmol/l], progesterone [nmol/l], total testosterone [nmol/l], dehydroepiandrosterone sulfate (DHEAS) [mcmol/l], sexual hormone binding globulin (SHBG) [nmol/l], thyroid stimulating hormone (TSH) [mU/l], free triiodothyronine (fT3) [pmol/l], free thyroxine (fT4) [pmol/l], antithyroid peroxidase autoantibodies (anti-TPO-AB) [IU/l], cortisol [nmol/l], haemoglobin [g/l], total cholesterol (CH) [mmol/l], HDL-CH [mmol/l], LDL-CH [mmol/l], triglycerides [mmol/l], fasting glucose [mmol/l], fasting insulin [mU/l], HOMA-IR (calculated), ferritin [mcg/l], CRP [mg/l], 25(OH) vitamin D3 [mmol/l]

|  | Ze 450 (n=32) | | MHT (n=142) | | P-value |
| --- | --- | --- | --- | --- | --- |
|  | n | median [lq, uq] or n (%) | n | median [lq, uq] or n (%) |  |
| FSH [U/L] | 25 | 54.8 [46.2, 83.4] | 116 | 56.5 [30.0, 78.4] | 0.68 |
| LH [U/L] | 12 | 25.7 [21.8, 40.5] | 51 | 25.7 [7.9, 43.4] | 0.58 |
| PRL [ug/L] | 12 | 13.7 [11.8, 22.1] | 105 | 11.4 [8.1, 15.2] | 0.045 |
| Estradiol [pmol/L] | 24 | 40.0 [20.0, 168.0] | 111 | 88.0 [31.0, 225.0] | 0.21 |
| Progesterone [nmol/L] | 6 | 0.70 [0.30, 1.90] | 63 | 1.10 [0.60, 2.50] | 0.24 |
| Total testosterone [nmol/L] | 6 | 1.45 [0.56, 1.60] | 35 | 1.50 [1.20, 2.00] | 0.42 |
| DHEAS [umol/L] | 22 | 3.33 [2.38, 4.15] | 113 | 2.99 [1.83, 3.88] | 0.24 |
| SHBG [nmol/L] | 0 |  | 19 | 52.1 [43.5, 88.0] |  |
| TSH [mU/L] | 26 | 1.76 [1.01, 2.41] | 131 | 1.64 [1.25, 2.93] | 0.59 |
| fT3 | 18 | 4.22 [4.10, 5.10] | 63 | 4.34 [4.01, 4.80] | 0.53 |
| fT4 | 19 | 16.3 [15.5, 17.8] | 64 | 14.6 [13.1, 16.6] | 0.019 |
| Anti-TPO-AK | 16 |  | 90 |  | 1.00 |
| Negative |  | 11 (69%) |  | 63 (70%) |  |
| Positive |  | 5 (31%) |  | 27 (30%) |  |
| Cortisol [nmol/L] | 11 | 392 [348, 525] | 95 | 456 [315, 553] | 0.87 |
| Total cholesterol [mmol/L] | 29 | 5.75 [4.63, 6.19] | 105 | 5.43 [4.81, 5.93] | 0.41 |
| HDL-cholesterol [mmol/L] | 29 | 1.69 [1.32, 1.99] | 105 | 1.74 [1.51, 2.12] | 0.46 |
| LDL-cholesterol [mmol/L] | 28 | 3.20 [2.44, 4.16] | 106 | 3.08 [2.61, 3.64] | 0.53 |
| Triglyceride [mmol/L] | 29 | 0.99 [0.74, 1.91] | 106 | 0.93 [0.75, 1.43] | 0.29 |
| Fasting glucose [mmol/L] | 26 | 4.82 [4.49, 5.19] | 94 | 4.93 [4.64, 5.31] | 0.56 |
| Fasting insulin [mU/L] | 17 | 6.60 [5.00, 9.40] | 65 | 7.60 [4.50, 10.10] | 0.99 |
| HOMA index (calculated) | 17 | 1.40 [1.10, 3.50] | 61 | 1.60 [0.90, 2.50] | 0.83 |
| Hemoglobine [g/L] | 19 | 138 [135, 144] | 48 | 137 [130, 144] | 0.32 |
| Ferritin [ug/L] | 24 | 73.0 [41.5, 133.5] | 114 | 78.5 [55.0, 137.0] | 0.48 |
| CRP < 5mg/L | 11 |  | 54 |  | 0.17 |
| No |  | 3 (27%) |  | 6 (11%) |  |
| Yes |  | 8 (73%) |  | 48 (89%) |  |
| 25(OH)Vitamin D3 [mmol/L] | 20 | 33.0 [25.5, 63.5] | 110 | 66.5 [42.0, 84.0] | 0.008 |

Supplementary Table 1: Serum hormone levels and blood chemistry at baseline. N=total number of observations, n=number of non-missing observations.
